# Crystal structure of 2-azido-1*H*-imidazole-4,5-di­carbonitrile

**DOI:** 10.1107/S2056989015013444

**Published:** 2015-08-06

**Authors:** G. Kenneth Windler, Brian L. Scott, Neil C. Tomson, Philip W. Leonard

**Affiliations:** a PO Box 1663 MS C920, Los Alamos National Laboratory, Los Alamos, NM 87544, USA; b PO Box 1663 MS J514, Los Alamos National Laboratory, Los Alamos, NM 87544, USA

**Keywords:** crystal structure, 2-azido-4,5-di­cyano-1*H*-imidazole, hydrogen bonding

## Abstract

In the title compound, C_5_HN_7_, the nitrile and azido substituents are close to being coplanar with the central ring. Mol­ecules in the crystal are linked *via* an N—H⋯N hydrogen bond to a nitrile acceptor, forming a chain extending along the *c-*axis direction.

## Related literature   

For background to imidazole applications, see: Windaus & Vogt (1907 ▸); Katritzky *et al.* (2006 ▸); Epishina *et al.* (1967 ▸); Srinivas *et al.* (2014 ▸). For preparations, see: Sheppard & Webster (1973 ▸); Lu & Just (2001 ▸); Parrish *et al.* (2015 ▸).
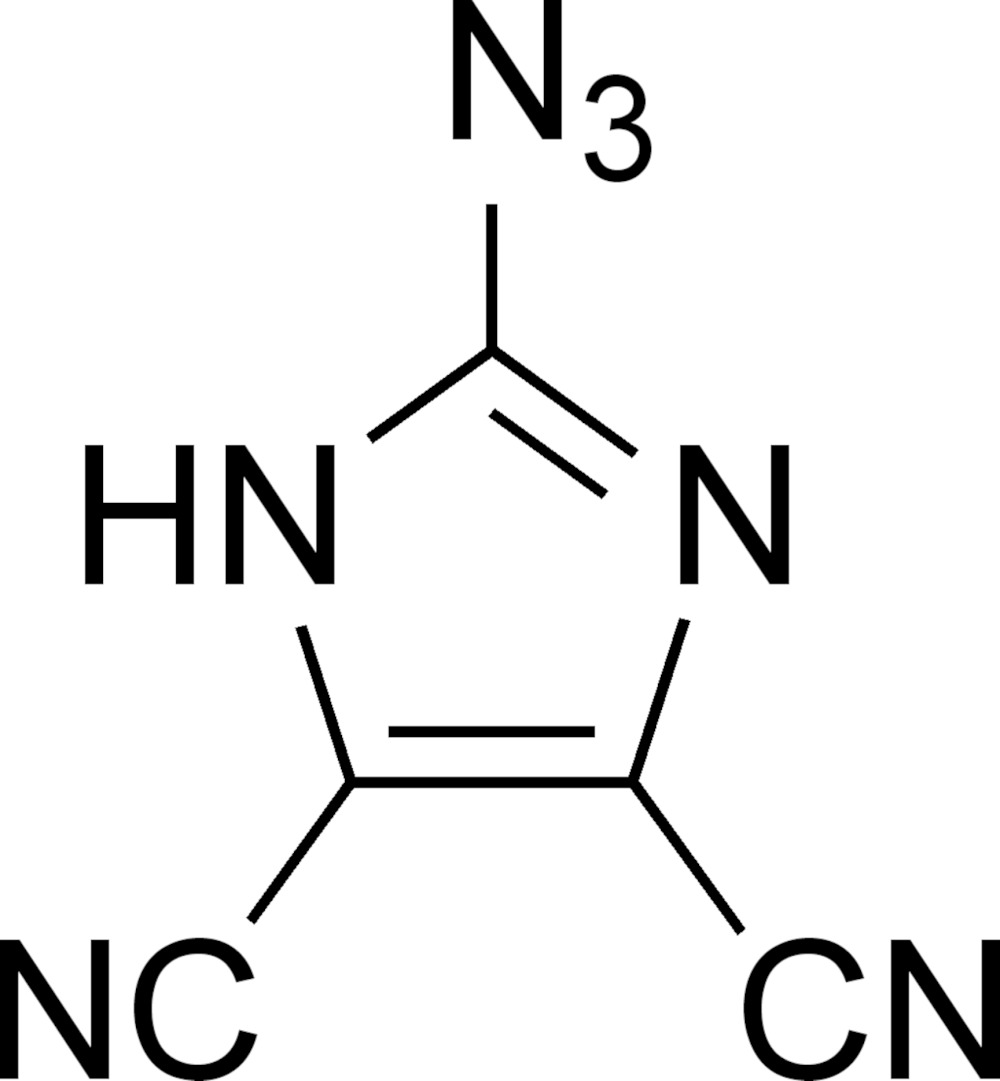


## Experimental   

### Crystal data  

C_5_HN_7_
*M* *_r_* = 159.13
Monoclinic, 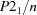

*a* = 7.3217 (6) Å
*b* = 12.8128 (11) Å
*c* = 7.5202 (6) Å
β = 102.215 (2)°
*V* = 689.51 (10) Å^3^
*Z* = 4
Mo *K*α radiation
μ = 0.11 mm^−1^
*T* = 100 K
0.36 × 0.24 × 0.10 mm

### Data collection  

Bruker D8 Quest with CMOS diffractometer
Absorption correction: multi-scan (*SADABS*; Bruker, 2009 ▸) *T*_min_ = 0.960, *T*_max_ = 0.989
13020 measured reflections
2943 independent reflections
2535 reflections with *I* > 2σ(*I*)
*R*_int_ = 0.024

### Refinement  

*R*[*F* ^2^ > 2σ(*F* ^2^)] = 0.036
*wR*(*F* ^2^) = 0.117
*S* = 1.56
2943 reflections
112 parameters
All H-atom parameters refined
Δρ_max_ = 0.51 e Å^−3^
Δρ_min_ = −0.25 e Å^−3^

### 

Data collection: *APEX2* (Bruker, 2009 ▸); cell refinement: *SAINT* (Bruker, 2009 ▸); data reduction: *SAINT*; program(s) used to solve structure: *SHELXS97* (Sheldrick, 2008 ▸); program(s) used to refine structure: *SHELXL97* (Sheldrick, 2008 ▸); molecular graphics: *ORTEP-3 for Windows* (Farrugia, 2012 ▸) and *Mercury* (Macrae *et al.*, 2008 ▸); software used to prepare material for publication: *CHEMDRAW Ultra* (Cambridge Soft, 2014 ▸).

## Supplementary Material

Crystal structure: contains datablock(s) I. DOI: 10.1107/S2056989015013444/zs2337sup1.cif

Structure factors: contains datablock(s) I. DOI: 10.1107/S2056989015013444/zs2337Isup2.hkl

Supporting information file. DOI: 10.1107/S2056989015013444/zs2337Isup3.cdx

Supporting information file. DOI: 10.1107/S2056989015013444/zs2337Isup4.cml

**Fig. 1.**
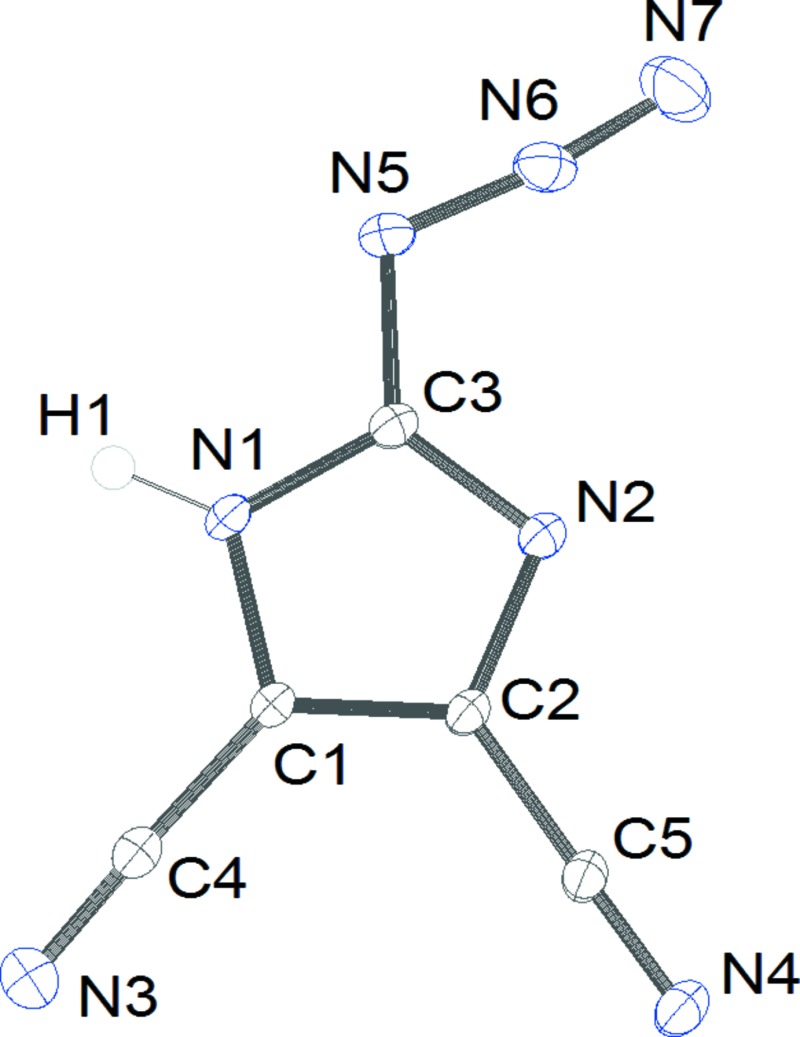
The molecular structure of the title compound with atom labeling. Ellipsoids are drawn at the 50% probability level, and the hydrogen atom is drawn as a sphere of arbitrary size.

**Fig. 2.**
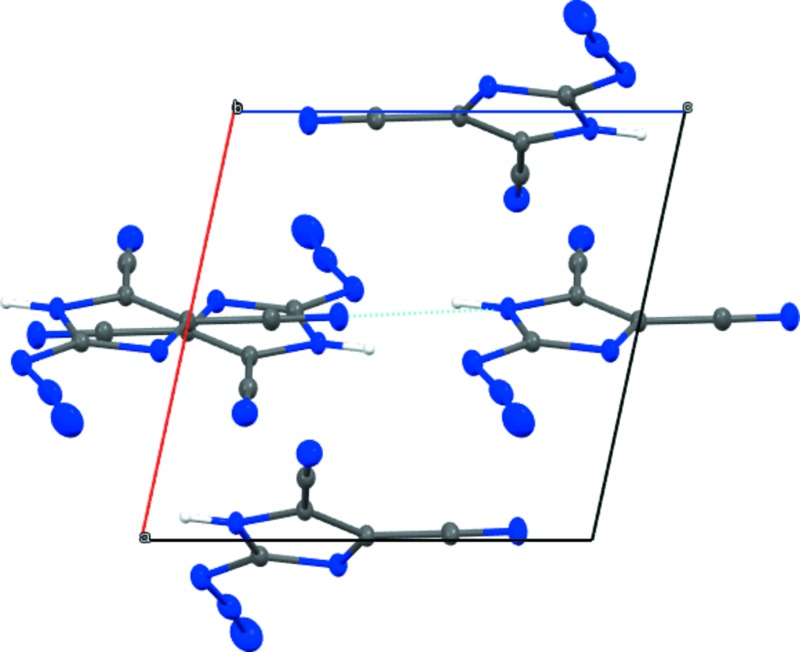
A crystal packing diagram of the title compound viewed along the b axis. The N—H···N hydrogen bond is shown as a dashed line.

. DOI: 10.1107/S2056989015013444/zs2337fig1.tif
The mol­ecular structure of the title compound with atom labeling. Ellipsoids are drawn at the 50% probability level, and the hydrogen atom is drawn as a sphere of arbitrary size.

b . DOI: 10.1107/S2056989015013444/zs2337fig2.tif
A crystal packing diagram of the title compound viewed along the *b* axis. The N—H⋯N hydrogen bond is shown as a dashed line.

CCDC reference: 1412579

Additional supporting information:  crystallographic information; 3D view; checkCIF report

## Figures and Tables

**Table 1:** Hydrogen-bond geometry (, )

*D*H*A*	*D*H	H*A*	*D* *A*	*D*H*A*
N1H1N4^i^	0.89(2)	2.00(2)	2.8572(9)	160.9(14)

Symmetry code: (i) 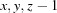.

## supplementary crystallographic information

### S1. Comment

Imidazoles have a storied history in the pharmaceutical (Windaus *et al.*, 1907), ionic liquid (Katritzky *et al.*, 2006), and energetic materials communities (Epishina *et al.* 1967). Recently, the title compound, C_5_HN_7_, appeared in a study of imidazoles as potential gas generators (Srinivas *et al.*, 2014). Given this background, we synthesized the title compound to examine the crystal structure, reported herein.

The entire molecule is essentially planar, with the maximum deviation indicated by the torsion angle in the ring atoms of 0.65 (7)° (C2—C1—N1—C3) and among the substituent groups, 176.76 (6)° (C3—N5—N6—N7) (Fig. 1). An intermolecular N1—H···N4 hydrogen bond involving a cyano N-atom acceptor (Table 1) generates a one-dimensional chain structure, extending along *c* (Fig. 2).

### S2. Experimental

To a stirred room temperature solution of sodium azide (4.40 g, 67.7 mmol) in water (100 ml) was added 2-diazo-4,5-dicyanoimidazole (4.05 g, 28.1 mmol) in portions (Sheppard & Webster, 1973; Lu & Just, 2001; Parrish *et al.*, 2015). Vigorous effervescence of liberated nitrogen gas occurred with each addition. The reaction was allowed to stir for a further 90 min after gas evolution ceased and was then extracted with ethyl acetate (4 *x* 20 ml). The organic layer was dried over magnesium sulfate and the solvent was removed by rotary evaporation to afford a light yellow solid. Crystals of the title compound suitable for X-ray diffraction were obtained by crystallization from ethyl acetate.

### S3. Refinement

The hydrogen atom was located in a difference-Fourier and the positional parameters were fully refined, with *U*_iso_(H) set invariant at 0.08.

### Crystal data

**Table:**

C_5_HN_7_	*F*(000) = 320
*M**_r_* = 159.13	*D*_x_ = 1.533 Mg m^−^^3^
Monoclinic, *P*2_1_/*n*	Mo *K*α radiation, λ = 0.71073 Å
Hall symbol: -P 2yn	Cell parameters from 2943 reflections
*a* = 7.3217 (6) Å	θ = 3.2–35.1°
*b* = 12.8128 (11) Å	µ = 0.11 mm^−^^1^
*c* = 7.5202 (6) Å	*T* = 100 K
β = 102.215 (2)°	Block, pale yellow
*V* = 689.51 (10) Å^3^	0.36 × 0.24 × 0.10 mm
*Z* = 4	

### Data collection

**Table:**

Bruker D8 Quest with CMOS diffractometer	2943 independent reflections
Radiation source: fine-focus sealed tube	2535 reflections with *I* > 2σ(*I*)
Bruker Triumph curved graphite monochromator	*R*_int_ = 0.024
ω scans	θ_max_ = 35.1°, θ_min_ = 3.2°
Absorption correction: multi-scan (*SADABS*; Bruker, 2009)	*h* = −11→11
*T*_min_ = 0.960, *T*_max_ = 0.989	*k* = −20→19
13020 measured reflections	*l* = −12→12

### Refinement

**Table:**

Refinement on *F*^2^	Primary atom site location: structure-invariant direct methods
Least-squares matrix: full	Secondary atom site location: difference Fourier map
*R*[*F*^2^ > 2σ(*F*^2^)] = 0.036	Hydrogen site location: inferred from neighbouring sites
*wR*(*F*^2^) = 0.117	All H-atom parameters refined
*S* = 1.56	*w* = 1/[σ^2^(*F*_o_^2^) + (0.0562*P*)^2^] where *P* = (*F*_o_^2^ + 2*F*_c_^2^)/3
2943 reflections	(Δ/σ)_max_ = 0.001
112 parameters	Δρ_max_ = 0.51 e Å^−^^3^
0 restraints	Δρ_min_ = −0.25 e Å^−^^3^

### Special details

**Table:**

Geometry. All e.s.d.'s (except the e.s.d. in the dihedral angle between two l.s. planes) are estimated using the full covariance matrix. The cell e.s.d.'s are taken into account individually in the estimation of e.s.d.'s in distances, angles and torsion angles; correlations between e.s.d.'s in cell parameters are only used when they are defined by crystal symmetry. An approximate (isotropic) treatment of cell e.s.d.'s is used for estimating e.s.d.'s involving l.s. planes.
Refinement. Refinement of *F*^2^ against ALL reflections. The weighted *R*-factor *wR* and goodness of fit *S* are based on *F*^2^, conventional *R*-factors *R* are based on *F*, with *F* set to zero for negative *F*^2^. The threshold expression of *F*^2^ > σ(*F*^2^) is used only for calculating *R*-factors(gt) *etc*. and is not relevant to the choice of reflections for refinement. *R*-factors based on *F*^2^ are statistically about twice as large as those based on *F*, and *R*- factors based on ALL data will be even larger.

### Fractional atomic coordinates and isotropic or equivalent isotropic displacement parameters (Å^2^)

**Table:**

	*x*	*y*	*z*	*U*_iso_*/*U*_eq_	
N1	0.95971 (8)	0.23656 (4)	0.20204 (8)	0.01244 (12)	
H1	0.946 (2)	0.2147 (12)	0.087 (3)	0.080*	
N3	0.79681 (9)	−0.00660 (5)	0.33122 (9)	0.01989 (14)	
N4	0.97866 (9)	0.20303 (5)	0.83076 (8)	0.01915 (14)	
N5	1.08587 (9)	0.39994 (5)	0.14988 (8)	0.01601 (13)	
N6	1.15492 (9)	0.48197 (5)	0.22731 (9)	0.01800 (14)	
N7	1.21954 (11)	0.55869 (5)	0.27860 (11)	0.02869 (17)	
N2	1.05744 (8)	0.33629 (4)	0.44841 (8)	0.01346 (13)	
C1	0.92901 (9)	0.17977 (5)	0.34810 (8)	0.01148 (13)	
C2	0.98919 (9)	0.24295 (5)	0.49778 (8)	0.01203 (13)	
C3	1.03672 (9)	0.32810 (5)	0.27026 (9)	0.01232 (13)	
C4	0.85397 (9)	0.07743 (5)	0.33564 (9)	0.01397 (13)	
C5	0.98406 (9)	0.22016 (5)	0.68201 (9)	0.01401 (14)	

### Atomic displacement parameters (Å^2^)

**Table:**

	*U*^11^	*U*^22^	*U*^33^	*U*^12^	*U*^13^	*U*^23^
N1	0.0156 (3)	0.0141 (3)	0.0079 (2)	0.00008 (18)	0.00303 (19)	0.00052 (17)
N3	0.0220 (3)	0.0172 (3)	0.0203 (3)	−0.0027 (2)	0.0043 (2)	−0.0015 (2)
N4	0.0240 (3)	0.0226 (3)	0.0118 (3)	−0.0040 (2)	0.0057 (2)	−0.0011 (2)
N5	0.0204 (3)	0.0149 (3)	0.0138 (3)	−0.00141 (19)	0.0059 (2)	0.00269 (19)
N6	0.0200 (3)	0.0162 (3)	0.0196 (3)	−0.0002 (2)	0.0082 (2)	0.0035 (2)
N7	0.0357 (4)	0.0186 (3)	0.0346 (4)	−0.0069 (3)	0.0137 (3)	−0.0007 (3)
N2	0.0162 (3)	0.0143 (3)	0.0104 (2)	−0.00187 (18)	0.00392 (19)	−0.00013 (18)
C1	0.0136 (3)	0.0123 (3)	0.0088 (3)	−0.0004 (2)	0.0029 (2)	0.00006 (19)
C2	0.0140 (3)	0.0136 (3)	0.0088 (3)	−0.0007 (2)	0.0033 (2)	−0.00039 (19)
C3	0.0132 (3)	0.0136 (3)	0.0107 (3)	0.0005 (2)	0.0036 (2)	0.0010 (2)
C4	0.0154 (3)	0.0159 (3)	0.0105 (3)	0.0004 (2)	0.0027 (2)	−0.0001 (2)
C5	0.0160 (3)	0.0154 (3)	0.0110 (3)	−0.0025 (2)	0.0038 (2)	−0.0021 (2)

### Geometric parameters (Å, º)

**Table:**

N1—C3	1.3545 (8)	N6—N7	1.1232 (9)
N1—C1	1.3752 (8)	N2—C3	1.3202 (8)
N1—H1	0.893 (19)	N2—C2	1.3770 (9)
N3—C4	1.1530 (8)	C1—C2	1.3808 (9)
N4—C5	1.1489 (9)	C1—C4	1.4171 (9)
N5—N6	1.2549 (8)	C2—C5	1.4241 (9)
N5—C3	1.3907 (8)		
			
C3—N1—C1	106.32 (5)	N2—C2—C1	111.15 (6)
C3—N1—H1	126.2 (11)	N2—C2—C5	121.75 (6)
C1—N1—H1	127.1 (10)	C1—C2—C5	127.10 (6)
N6—N5—C3	112.74 (6)	N2—C3—N1	113.73 (6)
N7—N6—N5	172.47 (8)	N2—C3—N5	128.17 (6)
C3—N2—C2	103.55 (5)	N1—C3—N5	118.09 (6)
N1—C1—C2	105.25 (6)	N3—C4—C1	177.65 (7)
N1—C1—C4	124.29 (6)	N4—C5—C2	179.07 (8)
C2—C1—C4	130.45 (6)		
			
C3—N1—C1—C2	0.65 (7)	C2—N2—C3—N5	−178.86 (7)
C3—N1—C1—C4	−178.28 (6)	N6—N5—C3—N1	179.67 (6)
C1—N1—C3—N2	−0.56 (8)	N6—N5—C3—N2	−1.31 (11)
C1—N1—C3—N5	178.61 (6)	N1—C1—C2—N2	−0.56 (8)
C3—N2—C2—C1	0.24 (8)	N1—C1—C2—C5	178.90 (7)
C3—N2—C2—C5	−179.26 (6)	C4—C1—C2—N2	178.28 (7)
C2—N2—C3—N1	0.20 (8)	C4—C1—C2—C5	−2.26 (12)

### Hydrogen-bond geometry (Å, º)

**Table:**

*D*—H···*A*	*D*—H	H···*A*	*D*···*A*	*D*—H···*A*
N1—H1···N4^i^	0.89 (2)	2.00 (2)	2.8572 (9)	160.9 (14)

Symmetry code: (i) *x*, *y*, *z*−1.
