# Supplementary figures and images for: Crystal structure of 2-azido-1H-imidazole-4,5-di­carbonitrile
Source: Acta Crystallogr E Crystallogr Commun. 2015 Aug 6;71(Pt 9):o633. doi: 10.1107/S2056989015013444 (PMC4555391; doi:10.1107/S2056989015013444)

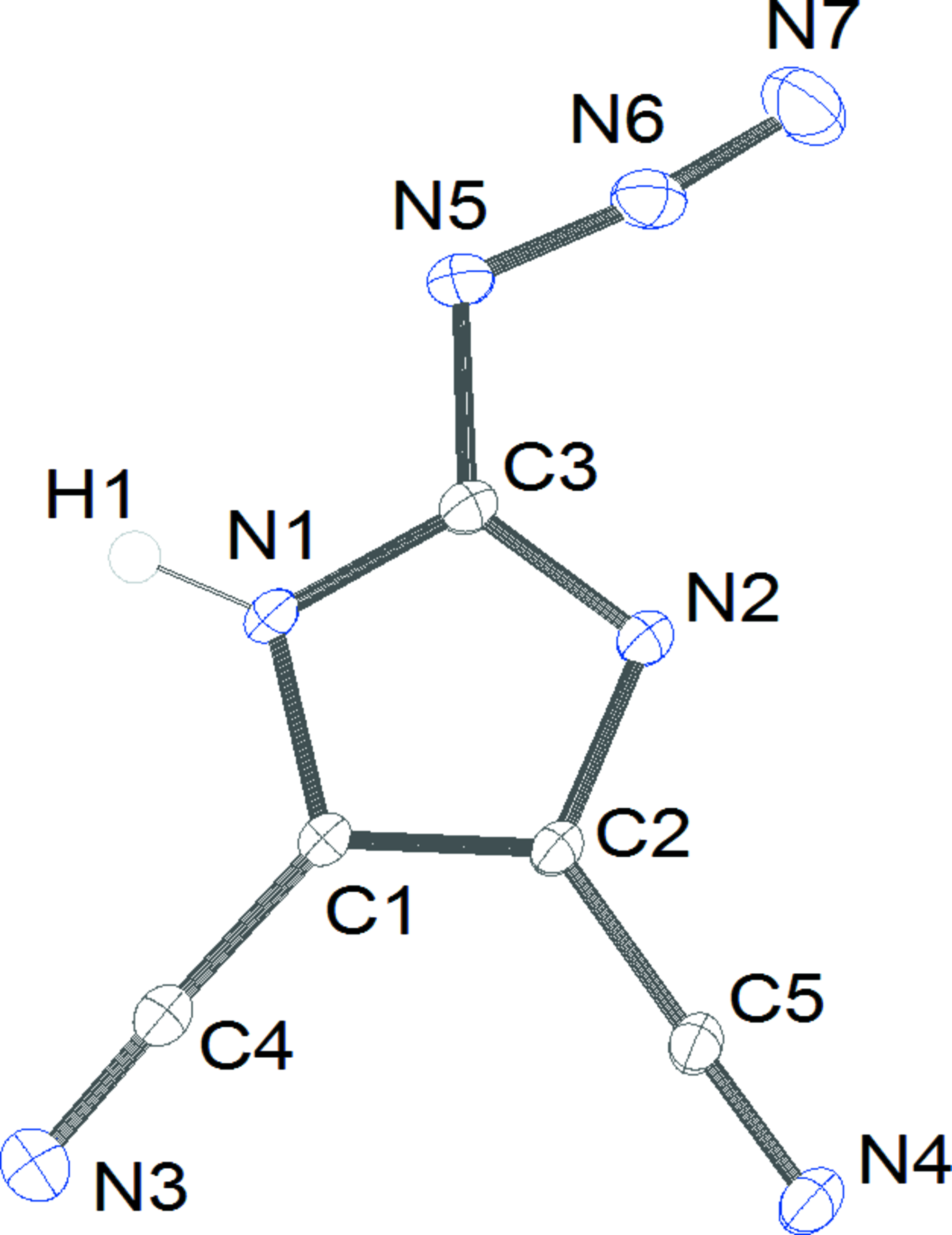

Supplement: Supplementary file 5 [file e-71-0o633-fig1.tif]

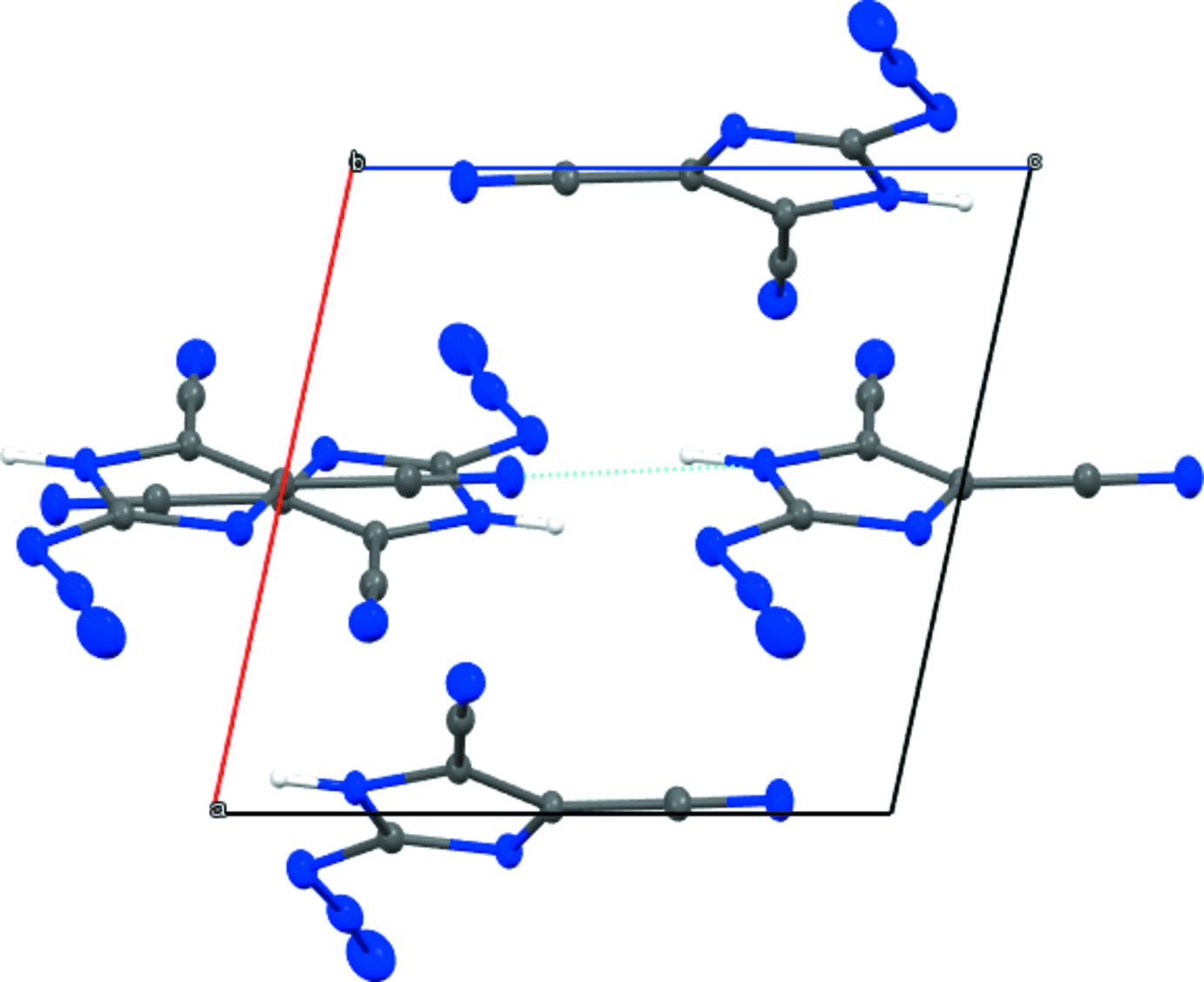

Supplement: Supplementary file 6 [file e-71-0o633-fig2.tif]
